# Weather Sensitivity of Sugar Bait Trapping of Nocturnal Moths: A Case Study from Northern Europe

**DOI:** 10.3390/insects13121087

**Published:** 2022-11-25

**Authors:** Nidal Fetnassi, Kadri Ude, Ain Kull, Toomas Tammaru

**Affiliations:** 1 Department of Zoology, Institute of Ecology and Earth Sciences, Faculty of Science and Technology, University of Tartu, 50409 Tartu, Estonia; 2 Water, Biodiversity and Climate Change Laboratory, Faculty of Sciences Semlalia, Cadi Ayyad University, P.O. Box 2390, Marrakech 40000, Morocco; 3 Department of Geography, Institute of Ecology and Earth Sciences, Faculty of Sciences and Technology, University of Tartu, 50410 Tartu, Estonia

**Keywords:** sugaring, Noctuidae, Erebidae, collecting, behaviour, adult feeding, thermal sensitivity, sweet baits, trap efficiency

## Abstract

**Simple Summary:**

Sugar baits are not only used to attract butterflies; they are also used to attract nocturnal moths. Similarly to most sampling techniques, the results of bait trapping are influenced by ambient conditions. In order to understand how bait trap catches depend on weather parameters, we performed a study in the north European forest zone using portable weather stations placed next to the traps. We found that the number of moths caught was strongly positively dependent on temperature and negatively dependent on air humidity. Diversity showed a similar dependence on temperature and humidity but was also negatively affected by air pressure and positively affected by the change of pressure during the night. Our findings help to interpret the results of quantitative insect trapping projects as they allow one to properly account for variability in weather conditions.

**Abstract:**

Assemblages of insects need to be quantitatively sampled in the context of various research questions. Light trapping is the most widely used method for sampling nocturnal Lepidoptera. Attracting moths to sugar baits offers a viable alternative. However, this method is rarely used in professional research despite its popularity among amateur lepidopterists. As the activity of insects is strongly dependent on ambient conditions, the sensitivity of any trapping method to weather parameters needs to be known for the quantitative interpretation of trapping results. In the present paper, we report data on the weather dependence of moth catches obtained by automatic bait traps. The study was performed in Estonia, representing the European hemiboreal forest zone. Portable weather stations set up next to each of the traps were used for collecting weather data. Both abundance and diversity of the moths in the catches depended strongly positively on temperature and negatively on air humidity. Diversity was also negatively correlated with air pressure and positively with the change in pressure during the night. The results show that in situ recording of weather parameters in connection to insect trapping provides useful insights for the study of insect behaviour and the interpretation of the results of monitoring projects.

## 1. Introduction

The need for sampling the assemblages of nocturnal Lepidoptera arises in various contexts ranging from basic faunistic research [1] and the use of moths as bioindicators of environmental changes [2,3,4] to monitoring projects of agricultural pests [5,6]. Attracting moths to artificial light sources (light trapping) has been the most widely used method for quantitative research on moths [7,8,9]. Best results can perhaps be obtained using automatic light traps with standardised designs [10,11]. Despite the possibility for a high degree of standardisation, light trapping is still inherently prone to yield biased results because of the unavoidable dependence of the catch on light conditions prevailing in the environment. For example, it is well known for lepidopterists that, under the conditions of the light summer nights of high latitudes, the traps are more effective in the forest compared to an open landscape just because it is darker there. On the other hand, forest vegetation can block the spread of light, making the net effect of these factors hard to evaluate. Similarly, the efficiency of light trapping is strongly dependent on moonlight and cloud cover [10,12]. Moreover, not all moth species are well attracted to light, which calls for the use of alternative sampling methods or combinations of different methods.

A viable alternative is offered by bait trapping, in which case adult lepidopterans are attracted to sugar-rich, typically fermenting substances representing or mimicking their natural food resources (sugar baits or sweet baits). Bait trapping is a common method in studies on tropical butterfly communities [13]. In contrast, automatic bait traps [14,15] appear to be relatively seldom used in quantitative studies on temperate moths. This is despite the popularity of sugar baiting among amateur lepidopterists, especially in Europe. Nevertheless, the number of studies based on bait trapping appears to be increasing in the context of research on various aspects of ecology [16,17,18,19] as well as pest management [20]. Sugar baits attract a different assemblage of species compared to light traps, and running a series of bait traps across the landscape is often logistically simpler since no power source is needed. Running bait traps is also less expensive compared to light traps. The flight activity of moths is strongly dependent on environmental conditions, which is a crucial factor to be considered in quantitative studies. For light traps, it has been demonstrated that the number of moths and species is influenced by various parameters such as temperature, wind speed and air humidity [10,11,12,21,22,23,24]. Not unexpectedly, the weather dependence of bait trap catches has been less extensively studied (see, however, [12]). According to amateur lepidopterist experience (personal observations of the authors and personal communication with numerous moth collectors), sugar bait catches are much less predictable than those of light trapping, holding the promise for non-trivial patterns to be discovered.

**Figure 1:**
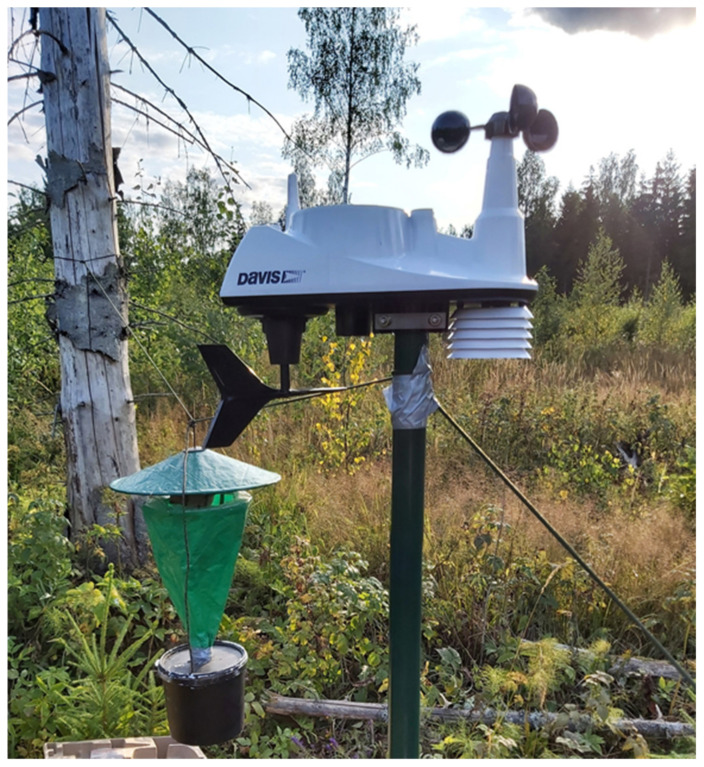
Data collection: a bait trap in an Estonian forest clearcut with a portable weather station next to it. The height of the metal pole is 1.5 m.

The aim of the present study is to contribute to the knowledge about the influence of weather parameters on bait trap catches, with the ultimate objective of providing guidance for the quantitative interpretation of bait trapping results in variable environmental conditions. Our study was performed in Estonia, representing the north European forest zone, and is based on data from 32 nights in the second half of summer 2021. A portable weather station was placed next to each trap, allowing for recording weather parameters in the immediate vicinity of the study site (Figure 1). The catches were collected the following morning, after which the traps were transferred to new locations.

## 2. Materials and Methods

The fieldwork was carried out on 32 nights over the period from 13 July to 2 September 2021 in the vicinity of the city of Tartu (58°23′ N, 26°43′ E; see Table 1 for exact locations), southern Estonia. The second half of summer was chosen for the study because this is the time of the year when bait trapping is most effective in the study area (personal experience of the authors).

On each night, 5 bait traps were simultaneously run in a study area throughout the scotophase. The study areas were represented by 4 clearcuts, which were 3 to 4 years old and situated 0.5 to 2 km from each other. When selecting clearcuts for the study, preference was given to those with altitudinal differences (max 20 m) to ensure some microclimatic diversity. All the clearcuts had an area of approximately 2 ha and were surrounded by hemiboreal mixed forests dominated by *Picea abies*, *Pinus sylvestris*, *Betula* spp., *Populus tremula* and *Alnus incana*. The vegetation on the clearcuts was primarily composed of a diverse assemblage of relatively tall (ca 0.5–1.5 m) herbaceous plants and some aftergrowth of the trees listed above, alternating with patches of dense growth of *Rubus idaeus*. As a consequence of forestry regulations in Estonia, all the clearcuts also harboured some retention trees [25].

The exact locations of the 5 traps within the clearcuts (labelled ‘site’ in the statistical analyses) were selected at about 100 m from each other; topographically different locations were preferred. The locations were not varied between different days of the study. Another study area (clearcut) was randomly chosen for the following night. As a result, all 4 clearcuts were sampled 8 times during the study period.

The traps (Tibiale insect equipment lc, Helsinki, Finland) consisted of a conical funnel made of foldable plastic (43 cm in length) covered by a circular roof (40 cm in diameter). The funnel fell into a plastic container with vapours of ethyl acetate as the killing agent (Figure 1; see [15] for a detailed description of a similar design). The bait constituted a piece of foam rubber (about 1 L in volume), with its lower part placed in a plastic cup, soaked in a mixture of beer and sugar (sucrose). A total of 1 kg of sugar was dissolved in 3 L of pale lager-type beer with 4.7% alcohol content (‘Saaremaa Tuulik’ by A. Le Coq). The bait was hanging from the roof in such a way that the upper surface of the foam was slightly above the upper margin of the funnel. Such traps rely on moths passively dropping from the bait through the funnel to the container after feeding. Sugar baits were numbered and rotated randomly between the sites each night to account for any possible effect of bait identity on the catch. The traps were hung on a rope drawn from the trunk of a retention tree to a 1.5 m tall metal pole hammered into the ground. The exact positions were chosen in such a way that access to the traps was not blocked by dense vegetation within a radius of about 2 m as a minimum. The weather stations (Vantage Vue Wireless, Davis Instruments Corporation, Hayward, CA, USA) were attached to the tops of the metal poles so that the distance from the trap to the station was about 1.5 m. The weather stations continuously recorded 10 weather parameters with the interval of 10 min. For the analyses, we used averages (or sums, for rainfall) of the weather parameters calculated alternatively from the period (1) from sunset to sunrise or (2) from sunset to midnight according to local solar time.

The moths were identified in the lab based on the morphological characters relying on the expertise of some of the authors; external experts were consulted when necessary. The total abundance (number of individuals) and Shannon index of diversity were calculated for each catch (combination of date and site), and these values were subsequently used as the dependent variables in the analyses. The effect of weather parameters on the third response variable, species richness (the number of species in nightly catches), was similar (Table S1) to the results for the Shannon index, and we chose not to consider this variable separately below.

General linear mixed models (GLMM) were built to analyse the data. In the main models, both the abundance and diversity of the moths were studied as dependent on predictors such as (nightly average) temperature, relative humidity, air pressure, wind speed and rainfall (see the tables in the Results section for details). Calendar date (and its square, to consider non-linearity) was used as a continuous covariate to account for phenological changes in moth assemblages, and ‘site’ (exact location of the trap within a clearcut) was included as a random effect. Another random factor, ‘bait identity’, did not attain significance in any of the analyses (based on the Wald test for covariance parameter estimates), so this variable was dropped from the models. We chose to fit normality-based models (with log-transformed values of abundance, the transformation being needed to normalise the distribution of residuals), whereas obvious overdispersion in the abundance data cautioned against applying models assuming Poisson distribution.

The initial models, including all the mentioned variables, were simplified through backward elimination to ensure that only statistically significant effects remained in the definitive models. The non-significance of the excluded variables was re-confirmed by adding them one by one to the definitive model in the final position (type I analysis was used). Such an approach was chosen to make sure that no variable was ‘lost’ in the backward elimination procedure due to collinearity. Collinearity was, nevertheless, not a major problem in our data (r < 0.5 for all such pairs of variables, which included one of the variables entering the definitive models, as presented in the tables in the Results section.

In the main analyses, all predictors were expressed as averages calculated over the entire scotophase of the trapping nights. However, alternatives were available as the weather parameters could also be expressed as the nightly maximal and minimal values, as well as the averages, minima and maxima for the first half of the night (when most moth activity is likely to occur). Accordingly, as a further step of the analyses, for both abundance and diversity, we tested which of the alternative ways of expressing temperature and humidity leads to the maximal predictive power of the model. As these alternative expressions of weather variables were strongly correlated with each other, we chose not to include them in the models simultaneously. Instead, the alternative measures of temperature and humidity were included in the definitive model one at a time, replacing the average values of temperature and humidity. Thereafter, the AIC values of the resulting models were compared to the AIC values of the main models.

Statistical analyses were carried out using the nlme package [26], R software version 4.1.2 (R Core Team, Vienna, Austria, 2021) [27].

## 3. Results

In total, 864 macroheteroceran moths from 59 species (primarily Noctuidae and Erebidae) were trapped during the 32 nights (=160 trap nights) of the study (Table 2). The variability in the weather parameters is presented in Table 3. Weather parameters such as temperature, air humidity and air pressure had a substantial effect on the catches, as discussed in detail below.

After removing the expected (it is trivial that moth assemblages change as the season progresses) effect of the calendar date, only temperature and humidity were found to affect the abundance (number of moths in the nightly catches). The effect of temperature was positive, whereas the effect of humidity was negative (fewer individuals were trapped in more humid conditions) (Table 4). The effect of air pressure, change in air pressure, wind and rain on abundance could not be confirmed (Table 5). Similarly to abundance, temperature was the main determinant of the diversity of the bait trap catches, with humidity also having a notable negative effect. In contrast to abundance, diversity was also affected by air pressure (lower diversity when air pressure was high) and the change in air pressure during the night (higher diversity when air pressure was increasing) (Table 6). The effect of wind speed and the occurrence of rain or dew did not attain significance (Table 5).

The analyses presented above relied on average values over the entire night. No alternative ways of expressing the variables lead to models differing from the basic one by more than 2 AIC units (Table 7). We, therefore, found no reason not to rely on the most straightforward solution, i.e., using nightly averages of the values of the weather parameters.

## 4. Discussion

In the present study, catches of automatic sugar (=sweet) bait traps of nocturnal Lepidoptera were studied as dependent on weather conditions prevailing during the trapping night. Differently from a number of previous studies addressing similar questions, weather parameters were recorded by portable weather stations placed in close vicinity to each of the individual traps. In addition to the frequently studied effects of temperature, humidity, wind and rainfall, we also addressed the often-neglected effect of air pressure. Statistical analysis of the effects of the weather parameters was performed after controlling for the phenological differences in moth abundance and diversity.

Not unexpectedly, temperature was revealed as the strongest determinant of both moth abundance and diversity. This agrees with the conclusions of most similar studies [12,23]. Indeed, for poikilothermic organisms like insects, average night temperatures are clearly suboptimal in the study area, with warmer nights always being more favourable for the activity of the adults. In quantitative terms, average moth abundance was about twice as high on the warmest nights than on the average nights of the study period, with the catches being close to zero on the coldest nights.

We found a negative effect of high air humidity on moth catches. For light traps, a similar result was reported by [23], while other studies [12,22,31] have found variable effects. We are unaware of works discussing biological reasons behind the possible effects of air humidity on the activity of adult insects. For light traps specifically, Jonason et al. [23] proposed that the frequent formation of fog on humid nights may reduce the attraction power of light traps. This explanation cannot obviously hold for bait traps, but our experience from the study area shows that the flight activity of moths on foggy nights is very low in general. The presence of night-time fog was not directly recorded in our study, but just a weak (and non-significant) effect of ‘dew’ (temperature dropping below the dewpoint) does not support ascribing the negative effect of high humidity to fog formation. Instead, biological factors specific to bait trapping may be involved: on humid nights, moths may more easily find alternative food sources and be less attracted to the baits. Moreover, moths may visit baits also just to avoid dehydration; the motivation of doing so should be lower under high humidity. Indeed, as some moths have been shown to modulate their feeding behaviour depending on the ambient humidity, consuming larger volumes of and more diluted nectar under low humidity conditions [32], attraction to liquid baits should be stronger on nights with low humidity.

No effect of wind was detected in our study. This contrasts several works reporting light trap catches decreasing with increasing wind speed ([12] and references therein, but see also [23,33]). It appears indeed obvious that, starting from a certain limit, strong winds should start disturbing insect flight, which cannot fail to negatively affect trapping efficiency. On the other hand, no air movement at all should have a negative effect on the efficiency of bait traps as air movement contributes to the spread of the smell of the bait, so a non-linear effect (optimal wind speed) is expected. Our study period was characterised by very weak winds in general, so the limit when winds start to disturb moths was likely not reached. A similar rationale may hold for the absence of the effect of rainfall in our analyses—there was no rain heavy enough during the study period to negatively affect moth flight performance (compare Table 3 for long-term averages).

The effect of air pressure on the efficiency of traps and insect behaviour in general appears not to have received much research attention (see, however, [31,34]). It appears reasonable to assume that insect responses to air pressure, and perhaps more specifically to changes in air pressure, may have an anticipatory character, predicting changes in those weather parameters which have a more direct biological relevance [35,36,37]. We found a negative effect of air pressure on diversity in moth catches, which is not unexpected as such because moth catches are known to be low on clear summer nights frequently associated with periods of high pressure. Nevertheless, such nights are also frequently cool and humid, so the effect of air pressure might be an indirect one. However, in our analyses, we showed that the effect of air pressure also persists in a model in which the effects of temperature and humidity have been accounted for, and air pressure may thus have a direct effect on insect behaviour. In turn, the positive effect of change in air pressure was in an unexpected direction as moths are generally known to be most active before the advent of rainy weather, expected to be associated with dropping atmospheric pressure. Further studies are needed to resolve this apparent contradiction.

Summing up, we believe that we have shown that in situ recording of multiple weather parameters has the potential to provide data useful for determining the weather sensitivity of insect trapping, a piece of information essential for interpreting trap-based abundance data in various applied and ecological contexts. We also found that the associations of Shannon diversity with weather parameters were more clear than those of total abundance, likely because total abundances are sensitive to large error variance due to locally high abundances of particular species. Overall, augmenting our ability to quantitatively understand the results of bait trapping should contribute to the wider adoption of this underused method in insect monitoring projects.

## Figures and Tables

**Table 1:** Location of the study areas (forest clearcuts) in Estonia.

Village	Coordinates *	Altitude (m asl) **
Ivaste	58°08′05″ N 26°37′26″ E	165
	58°08′02″ N 26°38′08″ E	176
Lutike	58°07′28″ N 26°36′32″ E	163
	58°07′13″ N 26°36′29″ E	156

*—Coordinates of the approximate centre of the clearcut; **—Average altitude of the 5 trapping sites within each clearcut.

**Table 2:** Macroheterocera recorded in the bait trap samples. Systematics and nomenclature follows [28].

Species	Total Abundance
Drepanidae	
*Thyatira batis*	193
*Tethea or*	2
*Ochropacha duplaris*	5
Geometridae	
*Timandra comae*	1
*Idaea biselata*	4
*Idaea aversata*	1
*Scopula immutata*	1
*Xanthorhoe spadicearia*	1
*Ecliptopera silaceata*	4
*Ecliptopera capitata*	2
*Eupithecia icterata*	1
*Hypomecis roboraria*	1
*Hypomecis punctinalis*	1
*Epione repandaria*	3
*Cabera exanthemata*	1
Sphingidae	
*Deilephila elpenor*	2
*Deilephila porcellus*	1
Erebidae	
*Scoliopteryx libatrix*	8
*Rivula sericealis*	6
*Pelosia muscerda*	2
*Lithosia quadra*	1
*Schrankia costaestrigalis*	137
*Parascotia fuliginaria*	6
*Catocala fulminea*	25
*Catocala fraxini*	192
*Catocala nupta*	31
*Catocala pacta*	3
Noctuidae	
*Autographa gamma*	10
*Deltote pygarga*	1
*Amphipyra pyramidea*	2
*Amphipyra perflua*	20
*Amphipyra tragopoginis*	1
*Allophyes oxyacanthae*	54
*Acronicta cuspis*	1
*Acronicta auricoma*	8
*Acronicta rumicis*	26
*Caradrina morpheus*	1
*Dypterygia scabriuscula*	1
*Trachea atriplicis*	33
*Amphipoea fucosa*	1
*Amphipoea oculea*	1
*Photedes fluxa*	17
*Xanthia togata*	1
*Agrochola helvola*	2
*Agrochola lota*	1
*Enargia paleacea*	1
*Ammoconia caecimacula*	10
*Mniotype satura*	7
*Lacanobia thalassina*	1
*Lacanobia oleracea*	1
*Mythimna conigera*	1
*Mythimna impura*	1
*Ochropleura plecta*	1
*Noctua pronuba*	4
*Eurois occulta*	1
*Xestia c-nigrum*	5
*Xestia baja*	11
*Xestia xanthographa*	3
Nolidae	
*Meganola strigula*	1

**Table 3:** Descriptive statistics. Averages and extreme values of trap-specific nightly mean values.

Variable	Average	Minimum	Maximum	Average Norm *
*independent*				
Temperature (°C)	12.1	3.4	18.6	13.4 (3.4–27.5)
Humidity (%)	93.4	78.5	98.5	78 (87)
Air pressure (mbar)	1010.9	998.7	1020.7	1014.0
Change in pressure during the night (mbar)	−0.05	−4.5	2.4	n.a.
Wind speed (m/s)	0.10	0.00	1.21	0.16
Rainfall (mm)	0.56	0.00	8.4	2.54
*dependent*				
Abundance	5.43	0	52	
Shannon index	0.72	0	2.78	

* Average norm corresponds to the mean August value of the standard climatological period of 1991–2020. Temperature in brackets indicates night-time minimum temperature and maximum temperature, and humidity in brackets corresponds to night-time average humidity of standard climatological period. Mean wind speed of standard climatological period is modelled from standard measurement height (10 m above ground level) to study height (1.5 m above ground level) according to European Wind Atlas methodology [29,30] considering surface roughness.

**Table 4:** Abundance (individuals in a trap per night, log-transformed) as a function of weather variables, a glmm model with ‘site’ (exact trap location) as a random factor. Type I analysis; definitive model obtained by backward elimination is presented. Switching the order of humidity and temperature in the model did not lead to qualitatively different results.

Type 1 Tests of Fixed Effects					
Effect	ω^2^, % *	NumDF	DenDF	F Value	*p*
Date	16.1	1	135	42.88	<0.0001
Date^2^	10.1	1	135	10.11	0.0018
Temperature	22.4	1	135	96.92	<0.0001
Humidity	9.7	1	135	37.57	<0.0001

*—Semipartial omega-squares presented to characterise the share of variance accounted for by each of the independent variables; ω^2^ for the effect of ‘site’ (trap location) was 6.2%.

**Table 5:** Statistics associated with a selection of weather variables when added one by one to the definitive models for abundance (Table 4) and diversity (Table 6). These variables did not enter the definitive models but were omitted in the backward elimination procedure. The analyses in this table provide additional confirmation of the non-significance of respective effects.

Variable	Abundance			Shannon Index		
	t	*p*	Direction ^1^	t	*p*	Direction ^1^
Air pressure	−0.42	0.66		X	X	X
Air pressure change	−0.86	0.39		X	X	X
Wind speed	−1.76	0.08	Negative	−0.4	0.69	
Maximal wind speed	−1.21	0.22		0.77	0.44	
Dew ^2^	−1.24	0.21		−1.33	0.18	Negative
Rainfall ^3^	0.15	0.87		1.39	0.16	Positive
Raining time ^4^	0.27	0.78		1.27	0.2	
Rain in daytime ^5^	1.16	0.24		1.82	0.07	Positive

^1^—direction of the effect is shown for variables with *p* < 0.2; ^2^—a binary variable expressing whether temperature dropped below the dew point or not; ^3^—summed precipitation during the night; ^4^—duration of the rainfall during the night, recorded by 10 min intervals; ^5^—a binary variable expressing whether it was raining in the study area between 4 p.m. and sunset on the day preceding the sampling night.

**Table 6:** Shannon diversity index of nightly trap catches as a function of weather variables, a glmm model with trap location as a random variable. Type I analysis; definitive model obtained by backward elimination is presented. Switching the order of humidity and temperature in the model did not lead to qualitatively different results.

Type 1 Tests of Fixed Effects					
Effect	ω^2^, % *	NumDF	DenDF	F Value	*p*
Date	19.4	1	134	21.30	<0.0001
Temperature	12.2	1	134	62.14	<0.0001
Humidity	6.3	1	134	34.46	<0.0001
Air pressure	2.5	1	134	6.70	0.0107
Air pr. change	0.9	1	134	5.94	0.0161

*—Semipartial omega-squares presented to characterise the share of variance accounted for by each of the independent variables; ω^2^ for the effect of ‘site’ (trap location) was 3.2%.

**Table 7:** Comparison of alternative models to the main models for (1) moth abundance (Table 4) and (2) Shannon diversity (Table 6) based on Akaike Information Criterion. The values of average nightly temperature and humidity (used in the main models) were replaced by alternative measures of these variables (maxima and minima of the full night; averages, maxima and minima for the first half of the night), resulting in five alternative models.

Measure of Temperature and Humidity	Abundance, Delta AIC	Shannon, Delta AIC
*Full night*		
Maximal	31.26	13.5
Minimal	20.4	0.89
*First half of the night*		
Average	1.12	2.17
Maximal	23.59	4.14
Minimal	4.69	**−1.72**

## Data Availability

Data are available upon request.

## Supplementary Materials

The following supporting information can be downloaded at: https://www.mdpi.com/article/10.3390/insects13121087/s1, Table S1: Species richness in nightly trap catches as a function of weather variables; a glmm model with trap location as a random variable. The analysis is analogous to that for Shannon index, presented in Table 6.

## References

[1] Murillo-Ramos L., Sihvonen P., Brehm G., Ríos-Malaver I.C., Wahlberg N. (2021). A database and checklist of geometrid moths (Lepidoptera) from Colombia. Biodivers. Data J..

[2] Franzen M., Johannesson M. (2007). Predicting extinction risk of butterflies and moths (Macrolepidoptera) from distribution patterns and species characteristics. J. Insect Conserv..

[3] Bell J.R., Blumgart D., Shortall C.R. (2020). Are insects declining and at what rate? An analysis of standardised, systematic catches of aphid and moth abundances across Great Britain. Insect Conserv. Divers..

[4] Uhl B., Wölfling M., Fiedler K. (2022). Exploring the power of moth samples to reveal community patterns along shallow ecological gradients. Ecol. Entomol..

[5] Choi H.S., Kim G.J., Shin H.J. (2011). Biocontrol of moth pests in apple orchards: Preliminary field study of application potential for mass trapping. Biotechnol. Bioprocess Eng..

[6] Yao Q., Lv J., Liu Q.J., Diao G.Q., Yang B.J., Chen H.M., Tang J. (2012). An insect imaging system to automate rice light-trap pest identification. J. Integr. Agric..

[7] Muirhead-Thomson R.C. (1991). Trap Responses of Flying Insects.

[8] Raimondo S., Strazanac J.S., Butler L. (2004). Comparison of sampling techniques used in studying Lepidoptera population dynamics. Environ. Entomol..

[9] Brehm G. (2017). A new LED lamp for the collection of nocturnal Lepidoptera and a spectral comparison of light-trapping lamps. Nota Lepidopterol..

[10] Fayle T.M., Sharp R.E., Majerus M.E.N. (2007). The effect of moth trap type on catch size and composition in British Lepidoptera. Br. J. Entomol. Nat. Hist..

[11] Bjerge K., Nielsen J.B., Sepstrup M.V., Helsing-Nielsen F., Høye T.T. (2021). An automated light trap to monitor moths (Lepidoptera) using computer vision-based tracking and deep learning. Sensors.

[12] Yela J.L., Holyoak M. (1997). Effects of moonlight and meteorological factors on light and bait trap catches of noctuid moths (Lepidoptera: Noctuidae). Environ. Entomol..

[13] Freitas A.V.L., Iserhard C.A., Santos J.P., Carreira J.Y.O., Ribeiro D.B., Melo D.H.A., Rosa A.H.B., Marini-Filho O.J., Accacio G.M., Uehara-Prado M. (2014). Studies with butterfly bait traps: An overview. Rev. Colomb. Entomol..

[14] Süssenbach D., Fiedler K. (1999). Noctuid moths attracted to fruit baits: Testing models and methods of estimating species diversity. Nota Lepidopterol..

[15] Laaksonen J., Laaksonen T., Itämies J., Rytkönen S., Välimäki P. (2006). A new efficient bait-trap model for Lepidoptera surveys—The “Oulu” model. Entomol. Fenn..

[16] Nieminen M., Hanski I. (1998). Metapopulations of moths on islands: A test of two contrasting models. J. Anim. Ecol..

[17] Nieminen M., Rita H., Uuvana P. (1999). Body size and migration rate in moths. Ecography.

[18] Merckx T., Kaiser A., van Dyck H. (2018). Increased body size along urbanization gradients at both community and intraspecific level in macro-moths. Glob. Chang. Biol..

[19] Jonason D., Franzen M., Pettersson L.B. (2013). Transient peak in moth diversity as a response to organic farming. Basic Appl. Ecol..

[20] El-Sayed A.M., Heppelthwaite V.J., Manning L.M., Gibb A.R., Suckling D.M. (2005). Volatile constituents of fermented sugar baits and their attraction to lepidopteran species. J. Agric. Food Chem..

[21] Butler L., Kondo C., Barrows E.M., Townsend E.C. (1999). Effects of weather conditions and trap types on sampling for richness and abundance of forest Macrolepidoptera. Environ. Entomol..

[22] Steinbauer M.J., Haslem A., Edwards E.D. (2011). Using meteorological and lunar information to explain catch variability of Orthoptera and Lepidoptera from 250 W Farrow light traps. Insect Conserv. Divers..

[23] Jonason D., Franzén M., Ranius T. (2014). Surveying moths using light traps: Effects of weather and time of year. PLoS ONE.

[24] Niermann J., Brehm G. (2022). The number of moths caught by light traps is affected more by microhabitat than the type of UV lamp used in a grassland habitat. Eur. J. Entomol..

[25] Rosenvald R., Lõhmus P., Rannap R., Remm L., Rosenvald K., Runnel K., Lõhmus A. (2019). Assessing long-term effectiveness of green-tree retention. For. Ecol. Manag..

[26] Pinheiro J., Bates D., DebRoy S., Sarkar D., R Core Team (2021). nlme: Linear and Nonlinear Mixed Effects Models. https://CRAN.R-project.org/package=nlme.

[27] R Core Team R. (2021). A Language and Environment for Statistical Computing (Version 4.1.2).

[28] Jürivete U., Õunap E. (2020). Estonian Lepidoptera Catalogue.

[29] Troen I., Peterson E.L. (1989). European Wind Atlas.

[30] Jaagus J., Kull A. (2011). Changes in surface wind directions in Estonia during 1966-2008 and their relationships with large-scale atmospheric circulation. Est. J. Earth Sci..

[31] Hikisz J., Soszynska-Maj A. (2015). What moths fly in winter? The assemblage of moths active in a temperate deciduous forest during the cold season in Central Poland. J. Entomol. Res. Soc..

[32] Contreras H.L., Goyret J., von Arx M., Pierce C.T., Bronstein J.L., Raguso R.A., Davidowitz G. (2013). The effect of ambient humidity on the foraging behavior of the hawkmoth Manduca sexta. J. Comp. Physiol. A Neuroethol. Sens. Neural Behav. Physiol..

[33] Komatsu M., Kurihara K., Saito S., Domae M., Masuya N., Shimura Y., Kajiyama S., Kanda Y., Sugizaki K., Ebina K. (2020). Management of flying insects on expressways through an academic-industrial collaboration: Evaluation of the effect of light wavelengths and meteorological factors on insect attraction. Zool. Lett..

[34] Miao J., Guo P., Li H., Wei C., Liu Q., Gong Z., Duan Y., Li T., Jiang Y., Feng H. (2021). Low barometric pressure enhances tethered-flight performance and reproductive of the oriental armyworm, Mythimna separata (Lepidoptera: Noctuidae). J. Econ. Entomol..

[35] Pellegrino A.C., Peñaflor M.F.G.V., Nardi C., Bezner-Kerr W., Guglielmo C.G., Bento J.M.S., McNeil J.N. (2013). Weather forecasting by Insects: Modified sexual behaviour in response to atmospheric pressure changes. PLoS ONE.

[36] Zagvazdina N.Y., Paris T.M., Udell B.J., Stanislauskas M., McNeill S., Allan S.A., Mankin R.W. (2015). Effects of atmospheric pressure trends on calling, mate-seeking, and phototaxis of Diaphorina citri (Hemiptera: Liviidae). Ann. Entomol. Soc. Am..

[37] Austin C.J., Guglielmo C.G., Moehring A.J. (2014). A direct test of the effects of changing atmospheric pressure on the mating behavior of Drosophila melanogaster. Evol. Ecol..

