# Supplementary material for: Weather Sensitivity of Sugar Bait Trapping of Nocturnal Moths: A Case Study from Northern Europe
Source: Insects. 2022 Nov 25;13(12):1087. doi: 10.3390/insects13121087 (PMC9783685; doi:10.3390/insects13121087)
Supplement: Supplementary file 1 [file insects-13-01087-s001.zip › insects-1976223-supplementary.pdf]

Table S1. Species richness nightly trap catches as a function of weather variables, a glmm model with trap location as a random variable. The analysis is analogous to that for Shannon index, presented in Table 6.

Type 1 Tests of Fixed Effects					
Effect	ω^2, %*	NumDF	DenDF	F Value	*p*
Date	17.3	1	134	25.31	<0.0001
Temperature	12.0	1	134	59.00	<0.0001
Humidity	3.6	1	134	24.29	<0.0001
Air pressure	2.0	1	134	6.12	0.0146
Air pr. change	0.2	1	134	2.67	0.1043
